# Targeting Autophagy, Apoptosis, and SIRT1/Nrf2 Axis with Topiramate Underlies Its Neuroprotective Effect against Cadmium-Evoked Cognitive Deficits in Rats

**DOI:** 10.3390/ph16091214

**Published:** 2023-08-29

**Authors:** Hany H. Arab, Ahmed H. Eid, Rania Yahia, Shuruq E. Alsufyani, Ahmed M. Ashour, Azza A. K. El-Sheikh, Hany W. Darwish, Muhammed A. Saad, Muhammad Y. Al-Shorbagy, Marwa A. Masoud

**Affiliations:** 1 Department of Pharmacology and Toxicology, College of Pharmacy, Taif University, P.O. Box 11099, Taif 21944, Saudi Arabia; h.arab@tu.edu.sa (H.H.A.); s.alsofyani@tu.edu.sa (S.E.A.); 2 Department of Biochemistry, Faculty of Pharmacy, Cairo University, Cairo 11562, Egypt; 3 Department of Pharmacology, Egyptian Drug Authority (EDA)—Formerly NODCAR, Giza 12654, Egypt; drahmedhamdy2007@yahoo.com (A.H.E.); raniaa.yahia@yahoo.com (R.Y.); drmerro@yahoo.com (M.A.M.); 4 Department of Pharmacology and Toxicology, College of Pharmacy, Umm Al Qura University, P.O. Box 13578, Makkah 21955, Saudi Arabia; amashour@uqu.edu.sa; 5 Basic Health Sciences Department, College of Medicine, Princess Nourah bint Abdulrahman University, P.O. Box 84428, Riyadh 11671, Saudi Arabia; aaelsheikh@pnu.edu.sa; 6 Department of Pharmaceutical Chemistry, College of Pharmacy, King Saud University, P.O. Box 11451, Riyadh 11451, Saudi Arabia; hdarwish@ksu.edu.sa; 7 Department of Pharmaceutical Sciences, College of Pharmacy, Gulf Medical University, Ajman 4184, United Arab Emirates; dr.abdullatif@gmu.ac.ae; 8 Department of Pharmacology and Toxicology, Faculty of Pharmacy, Cairo University, Cairo 11562, Egypt

**Keywords:** topiramate, cadmium, Alzheimer, autophagy, apoptosis, glutamate

## Abstract

Cadmium is an environmental toxicant that instigates cognitive deficits with excessive glutamate excitatory neuroactivity in the brain. Topiramate, a glutamate receptor antagonist, has displayed favorable neuroprotection against epilepsy, cerebral ischemia, and Huntington’s disease; however, its effect on cadmium neurotoxicity remains to be investigated. In this study, topiramate was tested for its potential to combat the cognitive deficits induced by cadmium in rats with an emphasis on hippocampal oxidative insult, apoptosis, and autophagy. After topiramate intake (50 mg/kg/day; p.o.) for 8 weeks, behavioral disturbances and molecular changes in the hippocampal area were explored. Herein, Morris water maze, Y-maze, and novel object recognition test revealed that topiramate rescued cadmium-induced memory/learning deficits. Moreover, topiramate significantly lowered hippocampal histopathological damage scores. Mechanistically, topiramate significantly replenished hippocampal GLP-1 and dampened Aβ42 and p-tau neurotoxic cues. Notably, it significantly diminished hippocampal glutamate content and enhanced acetylcholine and GABA neurotransmitters. The behavioral recovery was prompted by hippocampal suppression of the pro-oxidant events with notable activation of SIRT1/Nrf2/HO-1 axis. Moreover, topiramate inactivated GSK-3β and dampened the hippocampal apoptotic changes. In tandem, stimulation of hippocampal pro-autophagy events, including Beclin 1 upregulation, was triggered by topiramate that also activated AMPK/mTOR pathway. Together, the pro-autophagic, antioxidant, and anti-apoptotic features of topiramate contributed to its neuroprotective properties in rats intoxicated with cadmium. Therefore, it may be useful to mitigate cadmium-induced cognitive deficits.

## 1. Introduction

Cadmium is a prominent environmental contaminant that exists in tobacco and various foods such as root crops, cereals, vegetables, and seafood. Due to its ubiquitous usage in industrial and agricultural activities and its extended biological half-life in humans, cadmium exposure has been considered unavoidable [1,2]. Growing evidence has revealed that repeated cadmium exposure triggers marked neurotoxicity, including memory deficits. In this regard, several epidemiological studies have affirmed the diminished cognitive ability upon repeated exposure to cadmium metal in humans [3].

The pathways that mediate the neurotoxic impact of cadmium remain elusive. However, multiple mechanisms have been proposed, including redox perturbations and excessive apoptosis/neuronal cell loss in the brain hippocampus [4,5,6]. In this regard, ample evidence has revealed that the hippocampus plays a central role in the learning process and memory. Hence, cognitive dysfunction and impaired memory and learning have been characterized as hallmark manifestations of cadmium-induced neurotoxicity [4,5]. Neuronal pro-oxidant events are vital in the pathogenesis of cadmium neurotoxicity. In perspective, cadmium triggers the depletion of neuronal antioxidants and aberrant reactive oxygen species (ROS) generation. In the same regard, cadmium-evoked neurotoxicity is also mediated by inhibition of the nuclear factor erythroid 2-related factor-2 (Nrf2)/heme oxygenase-1 (HO-1) cascade. Intriguingly, there have been conflicting studies on the in vivo impacts of cadmium on Nrf2/HO-1 axis where activation [6], as well as inhibition [4] of this antioxidant cascade, have been characterized. Hence, further investigation is warranted to explore its exact role in the in vivo pathogenesis of cadmium neurotoxicity. In the same regard, the depletion of silent information-regulated transcription factor 1 (SIRT1) protein was observed in rodent models of cadmium-induced cognitive dysfunction [5]. In experimental animals, SIRT1 is essentially a cytoprotective NAD^+^-dependent deacetylase that dampens neuronal oxidative damage and apoptosis, resulting in considerable neuroprotection [7]. Upon exposure to excessive neuronal stresses and oxidative stress, neurons undergo cell death through the mitochondrial apoptotic pathway as the prevalent pathway. This is characterized by excessive pro-apoptotic signals like Bcl-2-associated x protein (Bax) together with diminished anti-apoptotic markers [5,6,8], which culminate in hippocampal neuronal loss and associated impairment of memory and learning in rodents [5,9].

Autophagy has recently been identified as a key player in the pathophysiology of cadmium-evoked neurotoxicity and cognitive impairment. Classically, autophagy is a catabolic condition that has evolved to cleanse cells of misfolded proteins and damaged mitochondria, thereby favoring cellular survival [10]. In the in vitro studies, conflicting results have been described in cadmium-triggered neuronal toxicity, where inactivation [10,11,12] and stimulation [13] of autophagy events have been characterized in neuronal cells. In addition, studies have inadequately addressed the in vivo autophagy process in experimental animals [6]. Specifically, the present study examines the effects of repeated cadmium exposure on rat hippocampi, the main brain area for learning and memory formation [14], focusing on autophagy events. According to the evolving evidence [13], autophagy is positively regulated by the adenosine monophosphate-activated protein kinase (AMPK)-mammalian target of rapamycin (mTOR) cascade, facilitating multiple neurotoxic signal removal [14].

The link between cadmium exposure and cognitive decline has been highlighted. Preclinical studies have characterized compromised learning ability and memory impairment in rodents intoxicated with cadmium [4,5]. Moreover, cellular and molecular studies have revealed that cadmium triggers the deposition of amyloid plaques and crosslinked amyloid β-peptide (Aβ) aggregates in the brain of animals, particularly in the hippocampus area. Meanwhile, rodent models of cadmium-induced cognitive disruption demonstrated that cadmium administration causes phospho-tau (p-tau) deposition as neurofibrillary tangles [14]. Together, the amyloid plaques and neurofibrillary tangles instigate hippocampal synaptic damage, neuritic dystrophy, and neuronal cell death, resulting in the manifestation of cognitive decline, memory impairment, and Alzheimer’s (AD)-like symptoms [2].

**Figure 1:**
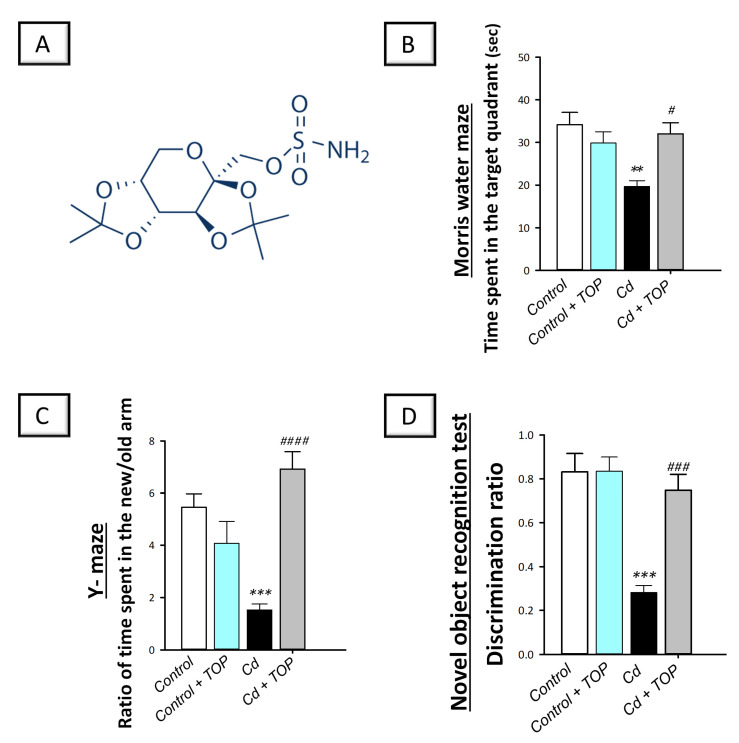
Topiramate counteracts the impairments in spatial learning/retention memory and recognition memory in cadmium-intoxicated rats. (**A**) Topiramate chemical structure. (**B**) The Morris water maze (MWM) test included 3 days of training (4 training sessions per day; 1 min each) where the hidden platform was placed in a fixed quadrant. Twenty-four h later, a probe test was executed where the hidden platform was removed. In the probe test, topiramate significantly increased the time spent in the target quadrant following platform removal, revealing an enhanced retention memory in animals. (**C**) As part of the Y-maze test (1 h after the training), animal’s short-term recognition memory was examined by measuring the ratio of the time spent in the new/old arm. In this test, the ratio was significantly increased by topiramate. (**D**) As part of the novel object recognition test (1 day after the training), animal’s long-term recognition memory was examined by measuring the discrimination ratio. In this test, the ratio was significantly increased by topiramate. In each group, *n* = 6 (graph presenting mean ± standard error of the mean). Statistical significance was denoted by ** p < 0.01 or *** p < 0.001, versus the control group. Statistical significance was denoted by *^#^ p* < 0.05, *^###^ p* < 0.001, or *^####^ p* < 0.0001, versus the cadmium group. TOP, topiramate; Cd, cadmium chloride.

As a sulfamate-substituted monosaccharide drug, topiramate serves as a tool for epilepsy management (Figure 1A shows its chemical structure). It elicits marked anti-epileptic actions by inhibiting the excitatory glutamate receptor subtype of 3-hydroxy-5-methyl-4-isoxazole-propionate (AMPA) and dampening the voltage-gated sodium channels, as well as increasing neurotransmission by aminobutyric acid (GABA) [15]. Beyond its classical use as an anti-epileptic drug, the clinical experience has suggested its efficacy for the management of psychiatric disorders, including bipolar disorders and essential tremors [16]. Meanwhile, it has been successfully used in schizophrenia-associated obesity and migraine [17]. Interestingly, topiramate has demonstrated remarkable neuroprotection against experimental models of focal as well as global cerebral ischemia in rodents [18] and postoperative cognition impairment in rats [19]. In the clinical setting, evidence revealed that topiramate has demonstrated notable efficacy in combating behavioral disturbances in patients with AD [16]. In this regard, several clinical trials have proven that topiramate is effective as a therapeutic approach for lowering the agitation and aggression behavior linked to AD dementia with similar efficacy to risperidone [16], carbamazepine, and valproic acid [20]. In the context of AD pathology, excessive excitotoxicity is involved in its pathogenesis [16,21]. Hence, dampening the hippocampal neuronal hyperactivity has been reported to enhance cognition, as reported in amnestic cognitive dysfunction [22]. Consistent with this notion, topiramate has been reported to suppress the activity of excitatory glutamate and augment the effects of GABA in neuronal cells, an event that may justify its efficacy against AD behavioral disturbance [15]. With respect to neurodegenerative diseases, topiramate has revealed marked neuroprotection against several experimental models, including 3-nitropropionic acid-induced striatal neurodegeneration, Huntington-like manifestations [23], and methylphenidate-triggered hippocampal neurodegeneration in the CA1 region and dentate gyrus of rats [24]. However, topiramate has not been examined in animals for its ability to combat learning and memory impairments triggered by cadmium. Therefore, the purpose of the present study was to assess whether topiramate may have a neuroprotective effect against cadmium-induced cognitive decline and the associated molecular and cellular manifestations. Herein, the cognitive impairment in terms of learning and memory dysfunction was investigated along with the molecular derangements and neurotoxic signals in the hippocampus of rats, the brain area that principally controls learning and memory activity [4,5,6]. In particular, the present work focused on neuronal autophagy, apoptosis, and redox changes.

## 2. Results

### 2.1. Topiramate Reverses Spatial Learning/Retention Memory Impairments Triggered by Cadmium

To explore whether topiramate can ameliorate cadmium-induced deficits in memory and spatial learning in vivo in animals, the Morris water maze (MWM) was employed to estimate rodent spatial memory and learning [25]. In the probe trial after removing the hidden platform, statistical significance was detected among groups [F (3, 20) = 6.873, *p* = 0.0023], as shown in Figure 1B. In comparison to the vehicle-treated control group, rats intoxicated with cadmium showed an impaired retention memory, with a significant (*p* < 0.01) reduction in the time spent in desired area by 42.4%. By administering topiramate to cadmium-intoxicated rats, this time was significantly (*p* < 0.05) increased by 62.9%. According to the MWM data, topiramate was able to improve the spatial learning/memory retention impairments caused by cadmium.

### 2.2. Topiramate Counteracts Cadmium-Induced Deterioration of the Recognition Memory in Rats

To examine whether topiramate can ameliorate cadmium-induced deterioration of the recognition memory in animals, the Y-maze was used as a reliable test for the spontaneous alternation behavior in rodents and recognition memory [26]. Regarding the time spent in the new/old arm ratio, statistical significance was detected among groups [F (3, 20) = 13.31, *p* < 0.0001] as shown in Figure 1C. Likewise, statistical significance was detected among groups in the discrimination ratio [F (3, 20) = 16.02, *p* < 0.0001] as shown in Figure 1D. In the Y-maze test, cadmium-intoxicated rats showed an impaired recognition memory in the short term (1 h post-training) as evidenced by the significantly (*p* < 0.0001) decreased ratio of the time spent in the new/old arm by 92.1%, versus the vehicle-treated control animals. In tandem, a significant (*p* < 0.001) decrease was detected in the discrimination ratio by 66.1% in the novel object recognition test, pointing to defective recognition memory in the long term (1 day). Topiramate administration to cadmium-intoxicated rats demonstrated an improvement in the recognition memory in the short and long term, as seen by the significant elevation of the ratio of the time spent in the new/old arm and the discrimination ratio, respectively. According to these data, topiramate was able to rescue the recognition memory impairment caused by cadmium.

### 2.3. Topiramate Ameliorates Hippocampal Neuronal Degeneration in Rats

**Figure 2:**
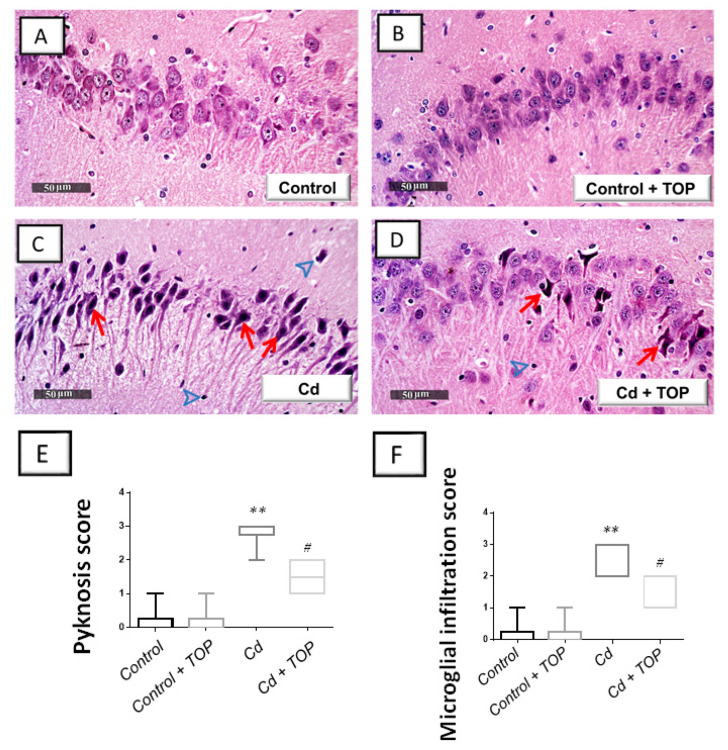
Topiramate attenuates hippocampal pyknosis and microglial cell influx in cadmium-intoxicated animals. Hematoxylin-eosin (H-E) staining of hippocampal sagittal sections was examined by light microscopy. In both the vehicle-treated control (**A**) and topiramate-treated control (**B**), intact subcellular and nuclear structures of the pyramidal neurons were revealed in the hippocampal region. (**C**) Cadmium intoxication triggered marked degenerative changes, including the pyknosis of pyramidal neurons (red arrow) and infiltration of microglial cells (arrowhead). (**D**) Topiramate administration to cadmium-intoxicated rats improved the hippocampal histological picture, as seen by lowered neuronal pyknosis (red arrow) and microglial cell influx (arrowhead) alongside an enhanced picture of intact neurons. (**E**,**F**) Significant lowering of pyknosis and microglial cell influx scores were observed in response to topiramate administration in cadmium-intoxicated animals. In each group, *n* = 6 (graph presenting median and interquartile range). Statistical significance was denoted by ** p < 0.01, versus the control group. Statistical significance was denoted by *^#^ p* < 0.05, versus the cadmium group. TOP, topiramate; Cd, cadmium chloride.

To examine whether topiramate can ameliorate cadmium-induced hippocampal histopathological aberrations and neuronal degeneration, the hippocampi of animals were examined by light microscopy. In both control and topiramate-treated control groups, normal hippocampal architecture was observed, characterizing normal pyramidal neurons showing intact subcellular details (Figure 2A,B). Cadmium intoxication triggered marked degenerative changes in the hippocampi of animals seen by pyknosis of pyramidal neurons and loss of neurons. In addition, the detection of moderate edema and infiltration of microglial cells were seen in the cadmium group (Figure 2C). Topiramate administration to cadmium-intoxicated rats demonstrated an improvement in the histology findings, as seen by the lowered neuronal pyknosis and microglial cell influx alongside the improved picture of intact neurons (Figure 2D). The pyknotic changes and microglial cell influx were quantified to further characterize the histologic aberrations. Herein, the pyknosis scores [H (3, 20) = 19.89, *p* = 0.0002] and microglial cell infiltration scores [H (3, 20) = 19.51, *p* = 0.0002] demonstrated statistical significance among groups as shown in Figure 2E,F. In comparison to the vehicle-treated control group, rats intoxicated with cadmium showed a significant elevation (*p* < 0.01) in the scores of pyknosis and microglial cell influx. By administering topiramate, there was a significant reduction in pyknosis scores (*p* < 0.05) and microglial cell influx scores (*p* < 0.05) by 47.1% and 50%, respectively.

### 2.4. Topiramate Decreases Hippocampal Neurodegeneration Signals in Cadmium-Intoxicated Rats

**Figure 3:**
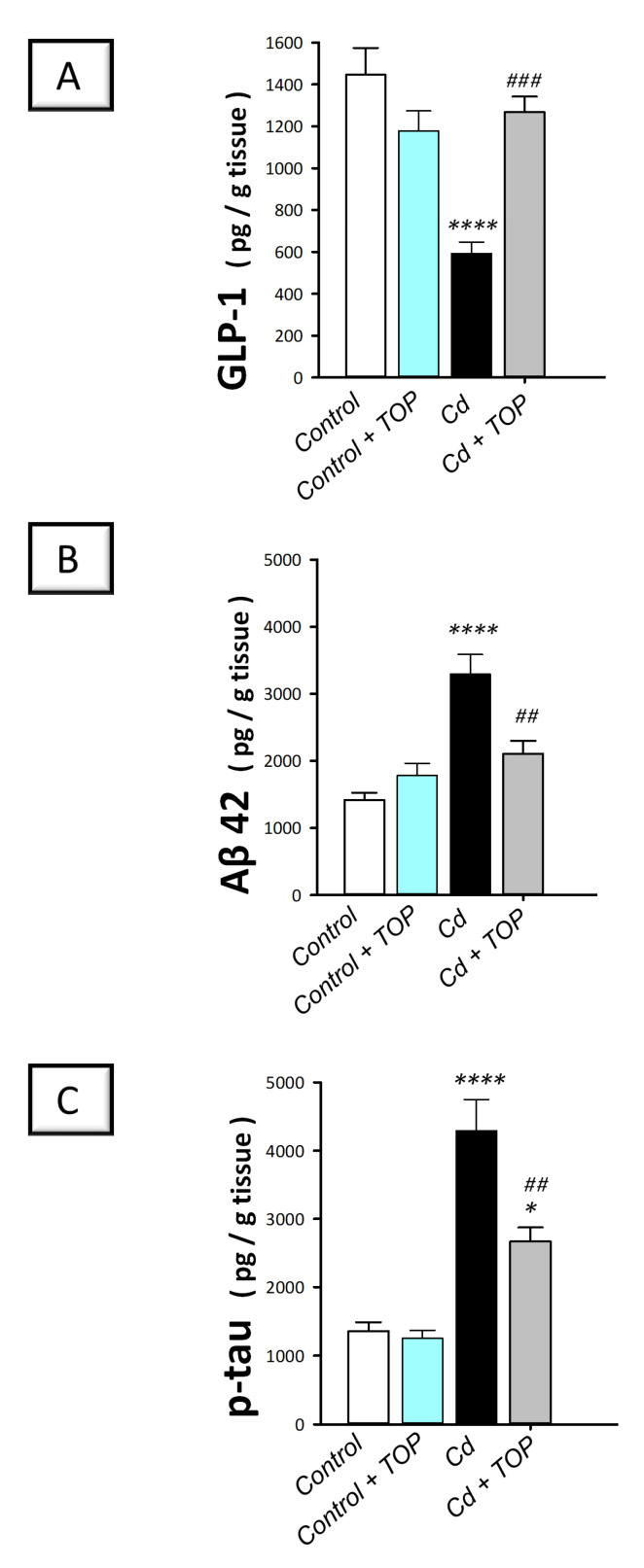
Topiramate increases hippocampal GLP-1 and lowers neurodegeneration signals in cadmium-intoxicated rats. Topiramate administration replenishes the protein expression levels of glucagon-like peptide-1 (GLP-1; (**A**)). Moreover, the neurodegeneration signals amyloid-beta 42 (Aβ42; (**B**)) and phosphorylated tau (p-tau; (**C**)) were diminished. In each group, *n* = 6 (graph presenting mean ± standard error of the mean). Statistical significance was denoted by * p < 0.05, or **** p < 0.0001, versus the control group. Statistical significance was denoted by *^##^ p* < 0.01, or *^###^ p* < 0.001, versus the cadmium group. TOP, topiramate; Cd, cadmium chloride.

To clarify the mechanisms involved in the pathology of cadmium-evoked neurotoxicity and Alzheimer-like molecular changes, animal hippocampi were examined to measure the neuroprotective glucagon-like peptide-1 (GLP-1) alongside the neurotoxic phosphorylated tau (p-tau) and amyloid-beta_1-42_ (Aβ_42_). Ample evidence demonstrated that elevated hippocampal levels of Aβ_42_ and p-tau are considered classical markers of the neuropathology of Alzheimer’s disease [2,14]. Herein, statistical significance was detected among groups in GLP-1 [F (3, 20) = 16.0, *p* < 0.0001], Aβ_42_ [F (3, 20) = 15.92, *p* < 0.0001], and p-tau [F (3, 20) = 28.53, *p* < 0.0001] as shown in Figure 3A–C. In comparison to the vehicle-treated control group, rats intoxicated with cadmium showed a significantly (*p* < 0.0001) lowered hippocampal GLP-1 by 59% alongside significantly (*p* < 0.0001) elevated levels of Aβ_42_ by 132.5% and p-tau by 216.9%. By administering topiramate to cadmium-intoxicated rats, significant augmentation was detected in hippocampal GLP-1 (*p* < 0.001) by 113.9%. Moreover, significant reduction was demonstrated in Aβ_42_ (*p* < 0.01) and p-tau (*p* < 0.01) by 35.9% and 37.8%, respectively. According to these data, topiramate was able to counteract Alzheimer-like neurotoxic signals by enhancing hippocampal GLP-1 alongside dampening Aβ_42_ and p-tau levels in cadmium-intoxicated rats.

### 2.5. Topiramate Rectifies the Neurotransmitter Changes in the Hippocampi of Cadmium-Intoxicated Rats

**Figure 4:**
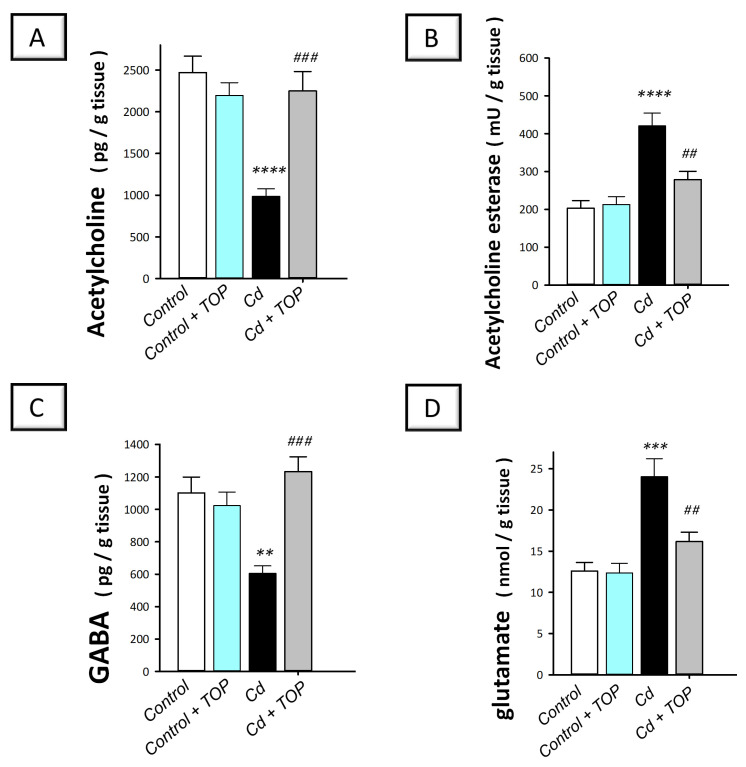
Topiramate improves the hippocampal neurotransmitter changes in cadmium-intoxicated rats. Topiramate administration replenishes the levels of acetylcholine (**A**) and γ-aminobutyric acid (GABA; (**C**)) and diminishes acetylcholine esterase activity (**B**) and glutamate levels (**D**). In each group, *n* = 6 (graph presenting mean ± standard error of the mean). Statistical significance was denoted by ** p < 0.01, *** p < 0.001, or **** p < 0.0001, versus the control group. Statistical significance was denoted by *^##^ p* < 0.01, or *^###^ p* < 0.001, versus the cadmium group. TOP, topiramate; Cd, cadmium chloride.

To explore the neurotransmitter changes associated with cadmium-induced neurotoxicity, the hippocampal levels of acetylcholine and its degrading acetylcholinesterase enzyme were measured. In fact, cholinergic transmission in the hippocampus has been tightly linked to cognitive decline [27]. Meanwhile, the levels of the inhibitory γ-aminobutyric acid (GABA) and the excitatory glutamate were determined. Herein, statistical significance was detected among groups in the levels of hippocampal acetylcholine [F (3, 20) = 14.32, *p* < 0.0001], GABA [F (3, 20) = 10.82, *p* = 0.0002], acetylcholine esterase activity [F (3, 20) = 16.23, *p* < 0.0001], and glutamate [F (3, 20) = 13.96, *p* < 0.0001] as shown in Figure 4A–D. In comparison to the vehicle-treated control group, hippocampal acetylcholine (*p* < 0.0001) and GABA (*p* < 0.01) were significantly lowered by 60.1% and 45%, respectively. Moreover, acetylcholine esterase activity (*p* < 0.0001) and glutamate levels (*p* < 0.001) were significantly elevated by 107.2% and 90.8%, respectively. By administering topiramate to cadmium-intoxicated rats, these neurotransmitter changes were reversed, as seen by a significant (*p* < 0.001) elevation in acetylcholine by 128.1% and GABA by 103.7%. This was accompanied by a significant (*p* < 0.01) reduction in acetylcholine esterase activity by 33.7% and glutamate by 32.7%. According to these data, topiramate’s ability to improve neurotransmitter changes is, at least partly, engaged in rescuing the cognitive decline associated with cadmium intoxication.

### 2.6. Topiramate Combats Hippocampal Redox Aberrations in Cadmium-Intoxicated Rats

**Figure 5:**
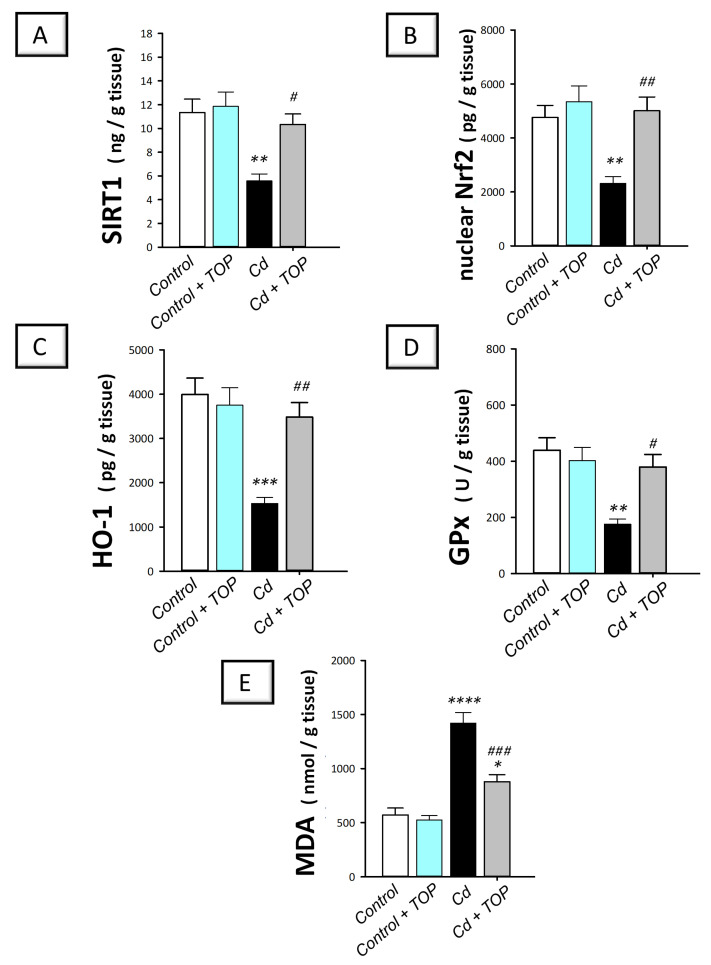
Topiramate curtails hippocampal redox aberrations in cadmium-intoxicated rats. Topiramate replenishes the antioxidant levels of SIRT1 (**A**), nuclear Nrf2 (**B**), HO-1 (**C**), and GPx (**D**) and diminishes the levels of the pro-oxidant malondialdehyde (MDA) (**E**) and in the hippocampi of cadmium-intoxicated rats. In each group, *n* = 6 (graph presenting mean ± standard error of the mean). Statistical significance was denoted by * p < 0.05, ** p < 0.01, *** p < 0.001, or **** p < 0.0001, versus the control group. Statistical significance was denoted by *^#^ p* < 0.05, *^##^ p* < 0.01, or *^###^ p* < 0.001, versus the cadmium group. Cd, cadmium chloride; TOP, topiramate; HO-1, heme oxygenase-1; GPx, glutathione peroxidase; Nrf2, nuclear factor erythroid 2-related factor-2; SIRT1, silent information-regulated transcription factor 1.

Neuronal degeneration and impairment of memory acquisition are tightly linked to the intensified oxidative events in the hippocampi of rodents [4,5]. Hence, the cytoprotective SIRT1 protein expression was examined. Meanwhile, the antioxidant Nrf2/HO-1 pathway and its downstream effector GPx were explored alongside the levels of lipid peroxides (MDA) [28,29]. Herein, statistical significance was detected among groups in the hippocampal levels of SIRT1 [F (3, 20) = 8.56, *p* = 0.0007], nuclear Nrf2 [F (3, 20) = 8.932, *p* = 0.0006], HO-1 [F (3, 20) = 12.11, *p* < 0.0001], GPx, [F (3, 20) = 8.52, *p* = 0.0008], and MDA [F (3, 20) = 34.51, *p* < 0.0001], as shown in Figure 5A–E. In comparison to the vehicle-treated control group, rats intoxicated with cadmium showed significantly (*p* < 0.01) reduced levels of the cytoprotective SIRT1 by 50.9%. Moreover, the hippocampi of cadmium-intoxicated animals revealed an excessive pro-oxidant response as indicated by a significant decline in HO-1 (*p* < 0.001) by 61.7%, nuclear Nrf2 (*p* < 0.01) by 51.4%, and GPx (*p* < 0.01) by 59.9% alongside a significant (*p* < 0.0001) elevation in MDA levels by 148.6%. By administering topiramate to cadmium-intoxicated rats, these pro-oxidant changes were reversed, as seen by a significant elevation in SIRT1 (*p* < 0.05) by 85.4%, HO-1 (*p* < 0.01) by 127.9%, Nrf2 (*p* < 0.001) by 116.1%, and GPx (*p* < 0.001) by 115.8% alongside a significant (*p* < 0.001) decline in MDA by 38.1%. According to these data, topiramate’s ability to suppress hippocampal oxidative events and stimulate SIRT1/Nrf2 axis is, at least partly, engaged in rescuing the cognitive decline associated with cadmium intoxication.

### 2.7. Topiramate Counteracts the Autophagy Impairment and Activates the Hippocampal AMPK/mTOR Cascade in Cadmium-Intoxicated Animals

**Figure 6:**
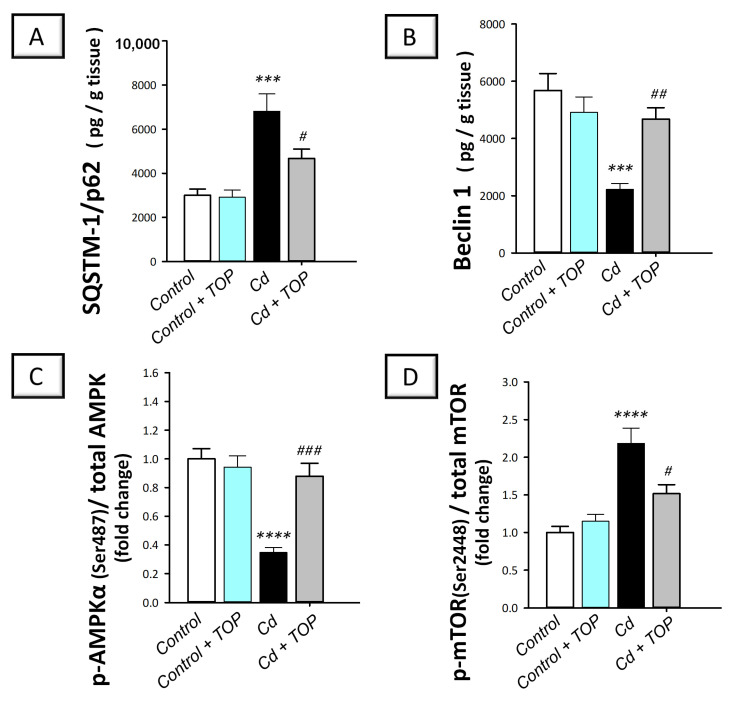
Topiramate ameliorates impaired hippocampal autophagy events in cadmium-intoxicated rats. This was evidenced by reduced SQSTM-1/p62 levels (**A**) and elevated Beclin1 (**B**). Moreover, topiramate administration to cadmium-intoxicated animals stimulated the AMPK/mTOR pathway, with an elevated p-AMPK/AMPK ratio (**C**) and lowered p-mTOR/mTOR ratio (**D**). In each group, *n* = 6 (graph presenting mean ± standard error of the mean). Statistical significance was denoted by *** p < 0.001, or **** p < 0.0001, versus the control group. Statistical significance was denoted by *^#^ p* < 0.05, *^##^ p* < 0.01, or *^###^ p* < 0.001, versus the cadmium group. AMPK, 5′adenosine monophosphate-activated protein kinase; Cd, cadmium chloride; mTOR, mammalian target of rapamycin; TOP, topiramate; SQSTM-1/p62, sequestosome-1/protein 62.

There is conflicting evidence regarding how cadmium affects autophagy in neurons in vitro. While studies have described stimulation of autophagy in hippocampal neuronal TH22 cells [13], other reports have described an impaired autophagy flux in PC12, primary murine neurons, and Neuro-2a cells [10,12]. In addition, a few studies have examined the in vivo impact of cadmium on hippocampal autophagy in rodents. Hence, hippocampal Beclin1 was examined alongside SQSTM-1/p62 as autophagy markers [14,30]. In addition, rat hippocampi were investigated for the pro-autophagic AMPK/mTOR pathway [31]. Herein, statistical significance was detected among groups in the levels of hippocampal Beclin 1 [F (3, 20) = 10.71, *p* = 0.0002], SQSTM-1/p62 [F (3, 20) = 13.69, *p* < 0.0001], p-mTOR/total mTOR ratio [F (3, 20) = 16.77, *p* < 0.0001], and p-AMPK/total AMPK ratio [F (3, 20) = 17.45, *p* < 0.0001], as shown in Figure 6A–D. In comparison to the vehicle-treated control group, a significant elevation was detected in SQSTM-1/p62 (*p* < 0.001) by 126.9% while a significant reduction was revealed in Beclin 1 by 60.9% (*p* < 0.001). Moreover, a significant increase was demonstrated in the p-mTOR/total mTOR ratio (*p* < 0.0001) by 118.4% alongside a significant reduction in p-AMPK/total AMPK ratio (*p* < 0.0001) by 65.3%. By administering topiramate to cadmium-intoxicated rats, the pro-autophagy events were stimulated as indicated by a significantly (*p* < 0.05) reduced SQSTM-1/p62 accumulation by 31.4% alongside a significantly (*p* < 0.01) elevated Beclin 1 by 110.6%. The autophagy-inhibitory signal p-mTOR/total mTOR was significantly (*p* < 0.05) decreased by 30.5% upon topiramate administration, while the p-AMPK/total AMPK ratio was significantly (*p* < 0.001) elevated by 153.2%. Accordingly, topiramate’s ability to stimulate the hippocampal autophagy events and the associated AMPK/mTOR cascade is, at least partly, engaged in rescuing the cognitive decline associated with cadmium intoxication.

### 2.8. Topiramate Curtails Hippocampal Apoptotic Cell Death in Cadmium-Intoxicated Rats

**Figure 7:**
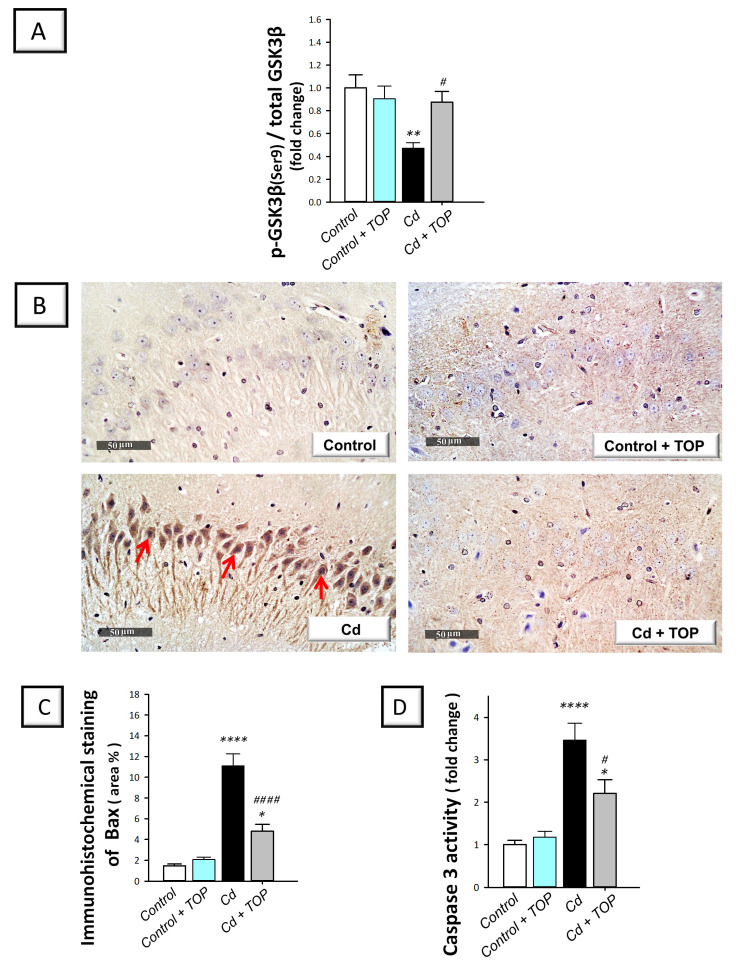
Topiramate lowers hippocampal apoptosis in cadmium-intoxicated animals. (**A**) The protein expression of p-GSK-3β(Ser9)/total GSK-3β. (**B**) Immunohistochemical staining of hippocampal Bax in rats (brown staining of the target protein is shown by red arrow; original magnification, 400×). (**C**) The graph displays Bax quantitative analysis (area %). (**D**) The activity of caspase 3. In each group, *n* = 6 non-overlapping fields (graph presenting mean ± standard error of the mean). Statistical significance was denoted by * p < 0.05, ** p < 0.01, or **** p < 0.0001, versus the control group. Statistical significance was denoted by *^#^ p* < 0.05, or *^####^ p* < 0.0001, versus the cadmium group. Bax, Bcl-2 associated x protein; GSK-3β, glycogen synthase kinase—3 beta; Cd, cadmium chloride; TOP, topiramate.

**Figure 8:**
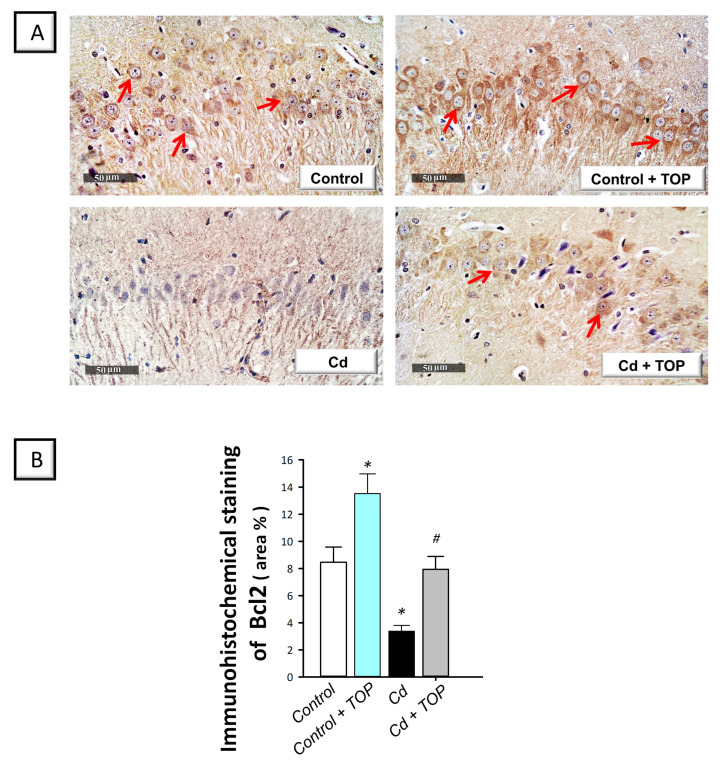
Topiramate increases hippocampal Bcl-2 protein expression in cadmium-intoxicated animals. (**A**) Hippocampal Bcl-2 immunohistochemical staining (brown staining of the target protein is shown by a red arrow; original magnification, 400×). (**B**) The graph displays Bcl-2 quantitative analysis (area %). In each group, *n* = 6 non-overlapping fields (graph presenting mean ± standard error of the mean). Statistical significance was denoted by * p < 0.05 versus the control group. Statistical significance was denoted by *^#^ p* < 0.05 versus the cadmium group. Cd, cadmium chloride; Bcl-2, B-cell lymphoma-2 protein; TOP, topiramate.

Ample evidence has demonstrated that defective neuronal autophagy is associated with the instigation of pro-apoptotic events in vitro [12]. Meanwhile, exaggerated hippocampal apoptotic cell death is associated with marked degeneration and behavioral deficits in cadmium neurotoxicity in rodents [5,6]. Thus, the executioner caspase 3, Bax, Bcl-2, and the upstream pro-apoptotic kinase GSK-3β were used as tools to examine hippocampal apoptosis. Herein, statistical significance was detected among groups in hippocampal p-GSK-3β(Ser9) [F (3, 20) = 8.588, *p* = 0.0007], Bax [F (3, 20) = 41.84, *p* < 0.0001], caspase 3 [F (3, 20) = 17.68, < 0.0001] (illustrated in Figure 7A,C,D), and Bcl2 [F (3, 20) = 15.40, *p* < 0.0001], as shown in Figure 8B. In comparison to the vehicle-treated control group, a significant decrease was demonstrated in the inactive form of GSK-3β (p-GSK-3β(Ser9); *p* < 0.01) and the anti-apoptotic Bcl2 (*p* < 0.05) by 52.8% and 60%, respectively, revealing excessive pro-apoptosis. Moreover, a significant elevation was shown in caspase 3 activity (*p* < 0.0001) and Bax protein (*p* < 0.0001) by 246.6% and 656.2%, respectively. By administering topiramate to cadmium-intoxicated rats, these pro-apoptotic changes were reversed, as seen by a significant elevation in p-GSK-3β(Ser9) (*p* < 0.05) and Bcl2 (*p* < 0.05) by 85.2% and 134.3%, respectively, alongside a significant lowering in Bax (*p* < 0.0001) and caspase 3 (*p* < 0.05) by 56.8% and 36.3%, respectively. According to these data, topiramate’s ability to suppress hippocampal apoptotic cell death and inactivate the pro-apoptotic GSK-3β is, at least partly, engaged in rescuing the cognitive decline associated with cadmium intoxication.

## 3. Discussion

**Figure 9:**
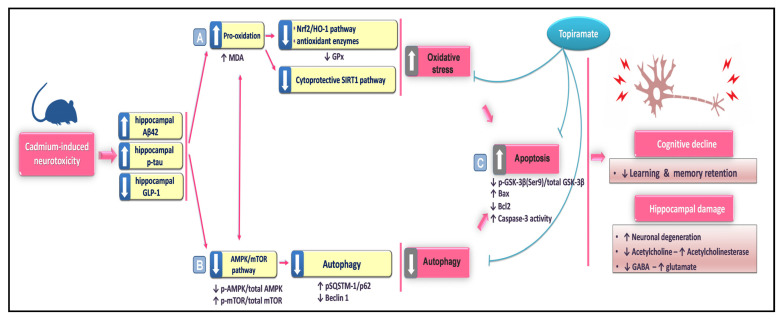
The mechanisms by which topiramate protected against cadmium-induced cognitive deficits. Herein, topiramate ameliorated cadmium-evoked memory and learning deficits by dampening hippocampal neurotoxic signals, including Aβ_42_ and p-tau and counteracting the neurtranmitter aberrations. These events were mediated by (A) activation of the hippocampal SIRT1/Nrf2/HO-1 cascade and attenuation of neuronal pro-oxidative events. (B) AMPK/mTOR pathway stimulation with an enhancement of the autophagy response. (C) Dampening of hippocampal apoptotic cell death. In the figure, solid arrows represent activation, whereas blunt arrows represent inhibition.

The current work discloses in vivo evidence for the protective effects of topiramate against cadmium-triggered cognitive impairment and Alzheimer’s (AD)-like neuropathology in rats. At the cellular and molecular levels, topiramate lowered hippocampal Aβ_42_ and p-tau, suppressing the excitatory glutamate and augmenting neuronal acetylcholine and GABA. Mechanistically, topiramate activated the hippocampal pro-autophagic events and dampened the oxidative insult and apoptotic machinery (Figure 9).

Ample evidence has characterized the cognitive deficits and AD-like neuropathology associated with repeated cadmium exposure [3]. In human epidemiological studies, higher cadmium levels were detected in postmortem specimens of AD patients relative to healthy controls [2]. In this context, cadmium can cross the BBB, resulting in notable accumulation in the brain hippocampus, the brain region that principally controls learning/memory tasks [4,5]. Keeping up with these data, the current work demonstrated that cadmium instigated deficits in spatial learning and recognition memory in animals, as seen in MWM, Y-maze, and NORT. This was accompanied by marked hippocampal accumulation of Aβ_42_ and p-tau neurotoxic signals, hallmark molecular manifestations of neuronal degeneration [4,5,6]. These events are reported to trigger neuronal death and synaptic disruption, culminating in memory impairment [5,14]. Moreover, these perturbations were accompanied by hippocampal activation of the neurotoxic signal GSK-3β, as seen by the lowered levels of the inactive p-GSK-3 (Ser9). The later signal contributes to AD pathogenesis by augmenting tau phosphorylation and Aβ_42_ production and suppressing acetylcholine production [32,33]. Herein, cadmium instigated hippocampal neurotransmitter aberrations, including a spike in the excitatory glutamate and a reduction in acetylcholine and GABA. In fact, excessive glutamate levels and associated activation of NMDA/AMPA receptors are involved in the pathogenesis of several neurodegenerative disorders [23], including AD [34]. Meanwhile, a decline in hippocampal cholinergic neurotransmission and cholinergic cell death have been previously reported in response to cadmium exposure [2,14].

Multiple lines of evidence have indicated that the modalities that can dampen hippocampal Aβ42 and p-tau neurotoxic signals and curb glutaminergic neurotransmission are associated with favorable attenuation of AD and associated behavioral deficits [27,35]. The present study demonstrated topiramate’s ability to mitigate cadmium-induced cognitive disruption that was revealed by lowering hippocampal Aβ_42_ and p-tau, suppressing glutamate levels, and the augmentation of acetylcholine and GABA content. Consistent with these findings, previous studies revealed that topiramate elicited marked neuroprotection against 3-nitropropionic-evoked striatal neurodegeneration and Huntington-like manifestations [23] and methylphenidate-triggered hippocampal neurodegeneration in the CA1 region and dentate gyrus of rats [24]. Notably, topiramate has been previously reported to lower Aβ_42_ production, tau phosphorylation, and GSK-3β activation [22].

In neurons, autophagy is involved in cellular homeostasis and synaptic plasticity, which is essential for memory acquisition and learning [10]. Impaired autophagy has been described in cadmium-induced neurotoxicity in vitro in rat primary cortical neurons [11], pheochromocytoma (PC12) [12], and Neuro-2a cells [10]. Likewise, defective autophagy has been described in preclinical animal models and postmortem brain samples of AD [36]. In this context, the impaired clearance of autophagic vacuoles in AD neurons leads to precipitation of amyloid aggregates [37]. Coinciding with these studies, the current findings revealed defective hippocampal autophagy in cadmium-induced cognitive deficit as manifested by a spike of SQSTM-1/p62 [14]. This event was corroborated with Beclin 1 decline, a marker for autophagosome synthesis at the level of sequestration step, affiriming autophagy impairment [36]. Indeed, the autophagy flux describes the sequential events of autophagosome production, delivery of the protein aggregates/damaged mitochondria to the lysosome, and finally, their destruction/recycling by lysosomal enzymes [36]. Notably, the observed impaired hippocampal autophagy may be paradoxical to an in vivo report that characterized autophagy activation in a cadmium-induced neurotoxicity model [13]. The contrast may be related to the difference in the length of cadmium exposure, animal species (rat vs. mouse), and the severity of hippocampal/cognitive damage [6,13].

Stimulation of autophagy has been proven as a key clearance pathway that removes neuronal aberrant Aβ aggregates [13] and p-tau/neurofibrillary tangles in AD [38]. In a preclinical model of AD, pharmacological activation of autophagy by rapamycin has been demonstrated to rescue behavioral deficits and tau neuropathology [13,14]. In the current experiments, topiramate instigated hippocampal SQSTM-1/p621 clearance, Beclin1 upregulation, and AMPK/mTOR pathway stimulation, pointing to notable autophagy activation. Indeed, topiramate’s pro-autophagic actions were previously characterized in transgenic mice [22] and metal-induced testicular deficits [39]. In neurodegenerative diseases, AMPK/mTOR pathway stimulation is associated with curbing the cognitive deficit and AD neuropathology by the removal of amyloid aggregates and p-tau [14]. In this context, the low-energy sensor AMPK can activate the autophagy flux by lowering the mTOR negative autophagy signal [4,14]. In several preclinical models of cognitive dysfunction, mTOR inhibition has been linked to marked neuroprotection. In the same regard, AMPK activators, such as resveratrol and quercetin, have been reported to enhance Aβ clearance, culminating in counteracting the neurotoxicity in rodents [14].

Upon exposure to increased levels of cadmium, neurons undergo apoptotic cell death mainly through the mitochondrial pathway [5,6]. Notably, the impairment of neuronal autophagy and buildup of autophagosomes instigates neuronal cell death in response to cadmium [40]. In this regard, a sequence of molecular events has been characterized when neurons are subjected to cadmium as a cell stressor. Initially, the autophagic events take place, then, apoptosis prevails when neuronal stress exceeds a critical duration/threshold [12,40]. Multiple lines of evidence revealed that cadmium-evoked cognitive decline is associated with enhanced hippocampal apoptosis with an increased Bax/Bcl-2 ratio [5,6,8]. The present findings supported earlier studies that cadmium-evoked cognitive deficit was linked to elevated hippocampal Bax, decreased Bcl-2, and activated GSK-3β. These apoptotic molecular derangements were counteracted by topiramate, culminating in a higher number of surviving neurons in the hippocampus and amelioration of memory deficits. Virtually, topiramate displayed marked anti-apoptotic features in preclinical models of 3-nitropropionic-induced Huntington-like disease [23] and methylphenidate-induced hippocampal damage [24]. These data were supported by the observed inactivation of GSK-3β, which advocates the anti-apoptotic features of topiramate. Indeed, GSK-3β inactivation exerts anti-apoptotic effects by upregulating Bcl-2 protein expression [33], an event that enhances the production of BDNF, a crucial signal for neuronal survival, differentiation, and protection [32]. Since neuronal oxidative stress is one of the major triggers for apoptosis, topiramate’s activation of the antioxidant SIRT1/Nrf2/HO-1 pathway can contribute to its anti-apoptotic actions [5,8]. Likewise, the observed and the reported [22] activation of autophagy events by topiramate also advocates the pro-survival signals by the removal of damaged mitochondria and ROS and associated clearance of aberrant Aβ aggregates [13] and p-tau/neurofibrillary tangles [38].

In response to glutamate flooding, excessive calcium influx takes place at the synaptic cleft culminating in superfluous ROS production and neuronal oxidative stress [23]. In the present study, cadmium augmented hippocampal glutamate, an event that was associated with SIRT1/Nrf2/HO-1 pathway inhibition and antioxidant depletion. These noxious events were reversed by topiramate administration. Consistently, marked antioxidant effects of topiramate have been characterized in 3-nitropropionic-evoked Huntington’s disease [23], where it boosted GSH and SOD and lowered MDA levels. Likewise, counteracting neuronal oxidative stress has mediated topiramate’s neuroprotective features in preclinical models of spinal cord damage [41] and methylphenidate-triggered hippocampal neurodegeneration [24]. In this regard, the antioxidant actions of topiramate have been ascribed to scavenging ROS, such as hydroxyl radicals and superoxide anions [42]. Notably, the observed hippocampal upregulation of SIRT1 by topiramate has been reported to counteract AD manifestations [7]. In perspective, upregulation of SIRT1 has been linked to dampened plaque precipitation and tau phosphorylation, culminating in the attenuated AD behavioral phenotype [7,43]. Moreover, SIRT1 augments Nrf2 transcriptional activity and the associated production of HO-1 and GPx antioxidant enzymes [6]. There is evidence that Nrf2 activation can ameliorate cadmium-evoked neurotoxicity in rodents and PC12 neuronal cells [44]. Notably, research has identified the crosstalk between SIRT1 and autophagy stimulation where SIRT1 activates the AMPK/mTOR pathway via dampening mTOR levels, affording neuroprotection in senile mice [45]. Likewise, the crosslink between cytoprotective Nrf2 and autophagy has been identified, where Nrf2 transcribes several autophagy genes, e.g., *LAMP2A* and *SQSTM-1/p62* [46].

The current study examined the neurochemical changes in the hippocampi of rats, the brain region that principally controls learning/memory tasks [4,5,6]. Consistent with this approach, rodents’ hippocampi have been examined in several studies to delineate the noxious effects of cadmium on cognition [5,13,27,47,48]. Of note, the memory function is also related to the nucleus basalis Mynert (NBM) in the basal forebrain, which provides cholinergic innervation to the cortex [49]. Previous studies have revealed that neuronal loss in NBM is associated with the loss of cortical cholinergic markers in AD. Interestingly, electrical stimulation of NBS elicits favorable outcomes on cognition by enhancing acetylcholine release, releasing multiple neuroprotective factors, promoting cerebral blood flow, and facilitating the cortical and subcortical receptive fields [50]. Regarding the effect of cadmium on NBM, the literature demonstrated that cholinergic neuron toxicity plays a crucial role in cadmium-induced deleterious effects on the brain. In this regard, cadmium exposure is associated with cholinergic neuron death in the basal forebrain [51]. An in vitro model of the basal forebrain using an SN56 cholinergic murine neuroblastoma cell line revealed that cadmium prompts apoptosis of basal forebrain cells, an event that was interceded by overexpression of Aβ and tau neurotoxic cues [2]. Recently, Sola and co-workers [52] reported that cadmium triggers neurodegeneration in rat basal forebrain—including the medial septal nucleus (MSN), the horizontal and vertical regions of the diagonal band of Broca (DBB), and NBM—by prompting thyroid hormone disruption. Another point to mention is that no positive control was used in the current study. This is due to the lack of specific FDA-approved drugs for combating the neurotoxic effects of cadmium. In the same regard, no previously shown agents—with comparable mechanism of action to topiramate—were demonstrated to dampen cadmium-induced cognitive decline in vivo. However, future studies using a positive control would be valuable in comparing the efficacy of the tested agent.

## 4. Materials and Methods

### 4.1. Drugs and Chemicals

Topiramate was received as a gift from Janssen-Cilag Pharmaceuticals (Raritan, NJ, USA). Sigma-Aldrich provided Cadmium chloride (Cat. # 202908; St. Louis, MO, USA). Under each determination, the reagent source is declared. All other chemicals were purchased at the highest purity possible.

### 4.2. Animals and Ethics

The directions of the Laboratory Animal Guide for Care and Use (US- NIH, Publication # 85-23, revised 1996, Bethesda, MD, USA) were applied regarding animal handling in the current protocol. An approval code NODCAR/I/7/2022 was issued by the Egyptian Drug Authority (EDA)’s Research Ethical Committee.

Forty Wistar albino rats (10-week-old, weighing 160–190 g) were utilized for the present study (The Breeding Unit of EDA, Giza, Egypt). Acclimatization was applied for two weeks, and during the whole study period, the animals were allowed free access to drinking water and laboratory food. Polycarbonate cages were used at the animal facility to house the animals at 21–24 °C, 50% humidity, and a 12 h darkness/light cycle.

### 4.3. Preclinical Animal Model

Rats were handled with care to reduce suffering and stress. Four experimental groups were used to carry out the experimental study (each group comprised 10 rats). With the aid of a blinded technician, the animals were randomly distributed, as shown in Table 1.

In the current study, the dose of cadmium chloride was selected on the basis of the previous literature [27,47,48]. In this context, a rat model of cadmium-evoked memory loss was successfully established using the 5 mg/kg/day dose of cadmium chloride. This was revealed by the declined spatial learning/memory function of animals in the Morris water maze in addition to the hippocampal neurodegenerative aberrations in histopathology [47]. The same dose was proven effective for incurring memory disruption and depleting acetylcholine in the hippocampi of rats [48]. Moreover, topiramate’s dose selection was established in accordance with earlier studies that showed it as beneficial for attenuation of 3-nitropropionic-induced striatal neurodegeneration and Huntington-like disease [23], methylphenidate-triggered hippocampal neurodegeneration [24], and pentylenetetrazol-induced epilepsy [53] in rodents.

This study demonstrated successful establishment of the animal model of cadmium-induced cognitive decline. This was revealed by the deterioration of spatial learning/retention memory in rats in the MWM and the impaired recognition memory as seen in the Y-maze and novel object recognition test (Figure 1B–D). Moreover, the histopathological findings further demonstrated the successful establishment of cadmium-induced neurotoxicity, which showed marked degenerative changes in the hippocampi of animals. These histopathological changes were quantified by the scores of neuronal pyknosis and microglial cell influx (Figure 2E,F). At the molecular levels, the observed cognitive decline was marked by increased hippocampal Aβ42 and p-tau neurotoxic signals (Figure 3) and aberrant neurotransmitter levels, including diminished hippocampal acetylcholine and GABA and elevated glutamate (Figure 4). Moreover, enhanced hippocampal oxidative stress (Figure 5) and apoptosis (Figure 7 and Figure 8), together with impaired autophagy (Figure 6), were detected in cadmium-intoxicated rats.

### 4.4. Morris Water Maze (MWM)

In this study, the MWM paradigm was used to study animals’ spatial learning and memory retention, as described earlier in [25]. A circular water tank (1.5 m diameter pool with 0.6 m height) was filled with water to 0.4 m depth. A 10 cm diameter platform was placed at the midpoint of the southeastern quadrant. The rats were trained for three days (four training sessions per day; 1 min each) to find the hidden platform, and the time spent locating the hidden platform was known as the escape latency time. A probe test (retrieval trial) was conducted on the fourth day after the platform was removed. In that test, the rat was allowed to explore the pool for 1 min. Measurement of memory consolidation was based on the time spent by the rat in the target quadrant to locate the hidden platform.

### 4.5. Y-Maze Test

Three identical arms were arranged at equal angles on the Y-maze, designated as A, B, and C. In terms of height and length, each arm measured 20 cm in height and 37 cm in length [32]. For the training session, one arm of the Y-maze was closed, and the session was applied for 10 min. Training in the apparatus involved positioning the animals in the center and allowing them to freely move through the open arms of the maze. The arm entries of each rat were visually observed. The test session was applied 1 h post-training, and the closed arm was opened. Herein, the recognition memory was examined in the short term (1 h post-training). In this context, good recognition memory is indicated by the more frequent entrance of the animal to the unexplored arm. During the study, the time spent in each arm was recorded, and the term “complete arm entry” refers to the animal entering the arm with all four paws. In the next step, we calculated the ratio of time spent in the new arm compared to the old arm. To avoid potential bias of animals due to olfactory cues, cleaning with 70% alcohol was applied after each trial/test.

### 4.6. Novel Object Recognition Test (NORT)

Using NORT, the recognition memory was evaluated in terms of the animal’s propensity for examining novel items. NORT’s arena measured 80 × 80 × 40 cm with a white background and black grid lines to act as a marking for the arena. The box was lighted in the experiment room, and no shadows were reflected on it. The NORT included three stages, namely (1) habituation, (2) familiarization (training), and (3) testing. During the habituation stage, animals were given 10 min to freely explore the empty box during the habituation process. After 24 h, the animals underwent a training (familiarization) stage. To this end, five minutes were given to the animal to explore two identical non-toxic objects (A1 and A2) placed in a fixed location. Exploration is reaching out with an animal’s nose for a distance less than or equal to 2 cm from an object. During the training session, animals that failed to investigate the objects were eliminated. After each session, 70% ethanol was used to clean the box and the items in order to eliminate any potential bias brought on by odor cues. In the testing process (24 h after the training), a novel object (B) with a similar material and color, but a different shape, was added instead of one of the well-known objects. Animals were then given 5 min to explore the novel object at their leisure. A video camera was right above the arena for the best view of the investigation. By dividing the amount of time spent examining a novel object by the sum of its time exploring both familiar and unfamiliar objects, the discrimination ratio was calculated [54].

### 4.7. Harvesting Brain Tissue

The animals were euthanized 24 h after the cognitive tests, and the blood was collected for serum separation. Immediately, brains were harvested for the isolation of hippocampi. The ELISA assays were performed using hippocampus homogenates prepared in protease/phosphatase-complemented lysis buffer (200 mM NaCl, 5 mM EDTA, 10 mM Tris, 10% glycerol). The homogenate supernatant was stored immediately at −80 °C for further processing after centrifugation. For histology and immunohistochemistry, four brains from each group were stored in 10% formalin-buffered saline.

### 4.8. Evaluation of Hippocampal Neurotransmitters, GLP-1, Aβ42, and p-tau

Hippocampal γ-aminobutyric acid (GABA) was measured with a specific ELISA kit (Cat. # E4457-100; BioVision Incorporated, Milpitas, CA, USA), and the hippocampal glutamate content was examined with an AFG Bioscience ELISA kit (Cat. # EK721805; AFG Bioscience, Northbrook, IL, USA). Concerning the acetylcholine/acetylcholine esterase axis, the activity of hippocampal acetylcholine esterase was determined with a specific ELISA kit (Cat. # KT-708; Kamiya Biomedical, Seattle, WA, USA) while the hippocampal acetylcholine content was measured using a Cloud-Clone Corp. ELISA kit (Cat. # CEA912Ge; Cloud-Clone Corp., Houston, TX, USA). A wavelength of 450 nm was used to determine the optical density.

A commercial ELISA kit was provided by SunLong Biotech Company in order to determine the concentration of glucagon-like peptide-1 in the hippocampus (GLP-1; Cat. # SL0304Ra; SunLong Biotech. Company, Ltd., Hangzhou, Zhejiang, China). The neurotoxic signals phosphorylated tau (p-tau) was determined with specific ELISA kit from Fine Test (Cat. #; ER1304; Fine Test, Wuhan Fine Biotech Co., Ltd., Wuhan, China) while the amyloid-β (Aβ42) was measured with a specific ELISA kit (Cat. # E-EL-R1402; Elabscience, Wuhan, China). A wavelength of 450 nm was used to determine the optical density.

### 4.9. Determination of the Pro-oxidant Markers

To quantify hippocampal lipid peroxides, the assay of thiobarbituric acid-reactive substance [55] was employed. A wavelength of 535 nm was used to determine the optical density. Under the instructions of the provider, a Sigma-Aldrich glutathione peroxidase (GPx) cellular activity test kit was used to detect GPx activity. This was achieved by monitoring the decline in absorbance at 340 nm using kinetic software. Regarding the SIRT1/Nrf2/HO-1 axis, the AFG Bioscience ELISA kit was used for the measurement of SIRT1 content (Cat. # EK720561; AFG Bioscience, Northbrook, IL, USA). Moreover, kits from AFG Bioscience (Cat. # EK720003; AFG Bioscience, Northbrook, IL, USA) and Elabscience (Cat. # E-EL-R0488; Elabscience, Wuhan, China) were used to quantify hippocampal Nrf2 and HO-1 target proteins, respectively. A wavelength of 450 nm was used to determine the optical density. Of note, the nuclear fraction extracts were analyzed for Nrf2 protein expression. This was achieved using a Cayman nuclear extraction kit (Cat. # MBS012148; Cayman Chemical, Ann Arbor, MA, USA), as guided by the provider.

### 4.10. Measurement of Apoptotic Events

In the study, we used commercially available ELISA kits to measure the ratio of p-GSK-3β(Ser9))/total GSK-3β. To this end, the total content of GSK-3β was quantified with a specific ELISA kit (Cat. #7265C) and the content of p-GSK-3β(Ser9) was assayed using a specific ELISA kit procured from Cell Signaling (Cat. # 7311C for GSK-3β(Ser9), Cell Signaling Technology, Danvers, MA, USA). An assay of hippocampal caspase 3 activity was performed with Sigma-Aldrich’s CASP-3-C colorimetric kit according to the manufacturer’s instructions (Sigma-Aldrich, St. Louis, MO, USA). A wavelength of 405 nm was used to determine the optical density.

### 4.11. Autophagy Events

Specific ELISA kits from AFG Bioscience (Cat. # EK720982; AFG Bioscience, Northbrook, IL, USA) and SunLong Biotech. (Cat. # SL1363Ra; SunLong Biotech. Company, Ltd., Hangzhou, Zhejiang, China) were procured to quantify Beclin1 and SQSTM-1/p62 protein expression, respectively, according to the manufacturer’s instructions. A wavelength of 450 nm was used to determine the optical density. Regarding the AMPK/mTOR pathway, the levels of p-AMPK(Ser487))/total AMPK and p-mTOR(Ser2448)/total mTOR were assayed using the commercially available kits. To this end, the RayBiotech ELISA kit was used to quantify the protein expression of p-AMPK(Ser487))/total AMPK (Cat. # PEL-AMPKA-S487-T; Norcross, GA, USA). In addition, the p-mTOR(Ser2448)/total mTOR ratio was investigated using the corresponding Cell Signaling Technology ELISA kit. In perspective, Cat. # 7974C was procured for total mTOR determination while Cat. # 7976C was utilized for p-mTOR(Ser2448) assay (Cell Signaling Technology, MA, USA) under the provider’s instructions. A wavelength of 450 nm was used to determine the optical density.

### 4.12. Histopathological Evaluation

To preclude bias, a technician/observer unaware of specimen identity performed the histology protocol. Prior to paraffin embedding in Paraplast embedding media, the formalin-fixed brain sections were washed, dehydrated with alcohol, and cleared in xylene [35]. Hematoxylin and eosin (H-E) staining of sections (5 µm thick) was performed for pathological evaluation. A light microscope was used to inspect the slides (Leica Microsystems GmbH, Wetzlar, Germany). In this study, four random specimens from each group were examined, and a total of six non-overlapping fields were captured for analysis [56].

Using the previously reported 0–4 scoring, the neuropathological damage for pyknosis and microglial cell infiltration was evaluated [57,58]. In the absence of specific lesions, a score of zero was assigned. On the other hand, in accordance with the affected area, neuropathological lesions were evaluated on a scale of 1 to 4 depending on the affected area: <10% (score of 1), 10–40%, (score of 2), 40–60% (score of 3), or >60% (score of 4). 

### 4.13. Immunohistochemical Evaluation

Using immunohistochemistry, the hippocampal protein expression of Bcl-2 and Bax was evaluated as characterized [36]. The de-paraffinized sections were processed for antigen retrieval, and a 3% H_2_O_2_ solution was used to block the endogenous tissue peroxidase. For tissue blockade, 5% bovine serum albumin was applied to the sections in a humidified chamber. For primary antibody incubation, sections of tissue were incubated at 4 °C overnight with anti-Bax (1:100 dilution; Cat. # 33-6600; Thermo Fisher Scientific, Fremont, CA, USA), or anti-Bcl-2 (1:100 dilution; Cat. # PA1-30411). The tissue slices were rinsed in PBS and treated for 20 min with an HRP-labeled secondary antibody (EnVision kit, Dako, Copenhagen, Denmark). Hematoxylin was applied for section counterstaining, and the target protein was immunostained with 3,3′-diaminobenzidine chromogen for 15 min. Light microscopy was applied to evaluate the immunohistochemical staining, and the total immunohistochemical staining area was calculated using the Leica Application module software (Leica Microsystems, GmbH, Wetzlar, Germany). Of note, the shown expression of Bax and Bcl2 target proteins has been validated for optimal staining as a routine process in the histopathology lab. Bias was precluded by keeping specimen identity confidential.

### 4.14. Statistical Analysis and Data Presentation

SPSS 17.0 software was applied to conduct statistical analysis (IBM, Chicago, IL, USA). The normal distribution of values was checked by the Shapiro–Wilk (parametric data). A one-way ANOVA test was used to compare parametric values, and multiple comparisons were conducted among all experimental groups using the Bonferroni test (at *p* < 0.05). On the other hand, the neuropathological damage scores (non-parametric) were processed by Kruskal–Wallis test and Dunn’s multi-comparisons. Plotting of figures was conducted using SigmaPlot (Systat Software, Inc., San Jose, CA, USA; version 12.0).

## 5. Conclusions

The cognitive deficits and AD-like neuropathology associated with cadmium were suppressed by topiramate in the present study. In perspective, topiramate curtailed hippocampal Aβ42 and p-tau neurotoxic signals, suppressed the excitatory glutamate, and augmented acetylcholine and GABA, culminating in behavioral recovery. Hence, the potential use of topiramate as an adjunct therapy to ameliorate cadmium-induced neurotoxicity seems promising. Virtually, this work serves as a proof-of-concept study that demonstrated the ability of topiramate to dampen cadmium-induced cognitive deficits in vivo in rats. Herein, the study mainly focused on the efficacy of topiramate to improve behavioral outcomes, including memory/learning deficits and histopathological aberrations, alongside some molecular events pertaining to hippocampal perturbations of redox milieu, apoptosis, and autophagy. However, further exploration of the detailed molecular mechanisms of topiramate is required using in vitro studies with an examination of the voltage-gated sodium channels and functional analysis of neurotransmitters as key targets for topiramate. Moreover, detailed quantification of the protein expression using Western blotting and double immunostaining is needed to elucidate topiramate’s molecular events. In addition, the effects of different doses of cadmium on behavioral tests in rats need to be further investigated.

## Figures and Tables

**Table 1:** Experimental design.

Group	*N*	Received
Control	10	Normal saline vehicle was orally received (10 mL/kg/day) by gavage. Two hours after normal saline administration, 0.5% carboxymethyl cellulose was given by oral gavage (10 mL/kg/day). A 2 h gap separated the administration of the two vehicles. The treatments lasted for eight weeks.
Control + TOP	10	Normal saline vehicle was orally received (10 mL/kg/day) by gavage. Two hours after normal saline administration, topiramate was given by oral gavage (50 mg/kg/day in CMC, delivered as 10 mL/kg/day). A 2 h gap separated the administration of the two doses. The treatments lasted for eight weeks.
Cd	10	Cadmium chloride solution (in normal saline; 5 mg/kg/day delivered as 10 mL/kg/day) was given by oral gavage. Two hours later, CMC was given by oral gavage (10 mL/kg/day). A 2 h gap separated the administration of the two doses. The treatments lasted for eight weeks.
Cd + TOP	10	Cadmium chloride solution (in normal saline; 5 mg/kg/day delivered as 10 mL/kg/day) was given by oral gavage. Then, topiramate (50 mg/kg/day suspended in CMC, delivered as 10 mL/kg/day) was also given by oral gavage 2 h after normal saline. A 2 h gap separated the administration of the two doses to avoid possible interaction. The treatments lasted for eight weeks.

## Data Availability

Data are contained within the article.
